# A Multi-Layer Classifier Model XR-KS of Human Activity Recognition for the Problem of Similar Human Activity

**DOI:** 10.3390/s23239613

**Published:** 2023-12-04

**Authors:** Qiancheng Tan, Yonghui Qin, Rui Tang, Sixuan Wu, Jing Cao

**Affiliations:** 1 College of Mathematics and Computing Science, Guangxi Colleges and Universities Key Laboratory of Data Analysis and Computation, Guilin University of Electronic Technology, Guilin 541004, China; 15878017512@163.com (Q.T.); ss_ysy@163.com (S.W.); 2 Center for Applied Mathematics of Guangxi (GUET), Guilin 541004, China; 3 Guangxi Key Laboratory of Automatic Detecting Technology and Instruments, Guilin University of Electronic Technology, Guilin 541004, China; 4 School of Advanced Manufacturing, Fuzhou University, Fuzhou 350108, China; 852102315@fzu.edu.cn; 5 College of Electrical Engineering and Information, Northeast Agricultural University, Harbin 150030, China; dnxtcj@163.com

**Keywords:** body-worn sensors, multi-layer classifier, random forest, kernel Fisher discriminant analysis, SVM, XGBoost feature selection algorithm

## Abstract

Sensor-based human activity recognition is now well developed, but there are still many challenges, such as insufficient accuracy in the identification of similar activities. To overcome this issue, we collect data during similar human activities using three-axis acceleration and gyroscope sensors. We developed a model capable of classifying similar activities of human behavior, and the effectiveness and generalization capabilities of this model are evaluated. Based on the standardization and normalization of data, we consider the inherent similarities of human activity behaviors by introducing the multi-layer classifier model. The first layer of the proposed model is a random forest model based on the XGBoost feature selection algorithm. In the second layer of this model, similar human activities are extracted by applying the kernel Fisher discriminant analysis (KFDA) with feature mapping. Then, the support vector machine (SVM) model is applied to classify similar human activities. Our model is experimentally evaluated, and it is also applied to four benchmark datasets: UCI DSA, UCI HAR, WISDM, and IM-WSHA. The experimental results demonstrate that the proposed approach achieves recognition accuracies of 97.69%, 97.92%, 98.12%, and 90.6%, indicating excellent recognition performance. Additionally, we performed K-fold cross-validation on the random forest model and utilized ROC curves for the SVM classifier to assess the model’s generalization ability. The results indicate that our multi-layer classifier model exhibits robust generalization capabilities.

## 1. Introduction and Related Work

Human activity recognition (HAR) involves identifying various human behaviors through a series of observations of individuals and their surrounding environment [1]. HAR has been generally applied in many fields, such as security and surveillance [2], sports and fitness [3], industry and manufacturing [4], autonomous driving [5], and the references therein.

A novel IoT-perceptive HAR approach based on a multi-head convolutional model was investigated in [6]. A hand-crafted and deep convolutional neural network feature fusion and selection strategy is given in [7]. In [8], the authors consider smart home environments using LSTM networks based on sensor-based smartphone data. In [9], a federated learning system with enhanced feature extraction was applied to HAR. The Bi-LSTM network was developed for multimodal continuous HAR [10]. In the field of industry and manufacturing, time factor analyses in conjunction with HAR have been considered for worker operating times [11]. As pointed out in [12,13], HAR technology improves the accuracy of targeting criminals with deep learning. The recognition of human activities has been applied to the development of a suitable autonomous driving system in the field of autonomous driving [14].

HAR methods can be broadly categorized into two main directions: vision-based HAR and wearable sensor-based HAR. It is well known that vision-based HAR has been concerned as well as wearable sensor-based HAR [15]. However, it faces several challenges, including privacy concerns related to potential video data leakage and the significant computational power and storage resources required for image processing. Additionally, factors such as the observer’s position and angle, the subject’s physique, background color, and light intensity can impact the accuracy of vision-based HAR [16]. In contrast, inertial sensor technology is typically cost-effective and offers greater robustness and portability in various environmental conditions [17]. Currently, sensor-based recognition technology has gained widespread attention due to its superior confidentiality and relatively lower computational requirements. The role of sensor placement in the design of HAR systems to optimize their availability has been discussed in [18]. Leveraging these advantages, wearable sensor-based HAR has garnered increasing interest in recent years.

The earliest research on sensor-based recognition of human behavior can be traced back to the 1990s, studied by researchers such as Foerster [19] and Bouten [20]. Nowadays, research on wearable sensors has resulted in the development of many highly accurate models. In [21], the authors achieved an overall accuracy of 84% by effectively collecting data and using decision tree classification. The Centinela system, developed by Lara and colleagues, achieved an overall accuracy of 95.7% [22]. However, a problem was identified where single-classification models can cause confusion when distinguishing similar activities, such as ascending stairs and descending stairs. In a study conducted by Jansi et al. [23], they utilized chaotic mapping to compress raw tri-axial accelerometer data and extracted 38 time-domain and frequency-domain features. These features included mean, standard deviation, root mean square, dominant frequency coefficient, spectral energy, and others. They achieved a recognition accuracy of 83.22% in human activity recognition. However, the results showed significant confusion between activities such as running, ascending stairs, and descending stairs. In the research conducted by Vanrell et al. [24], a 91-dimensional feature vector was extracted from single-axis accelerometer data. This vector included cepstral coefficients, time-domain features, and periodicity features. They achieved a recognition accuracy of 91.21% in a classification task involving ten different human activities. However, the results also indicated substantial confusion between activities such as cycling on an exercise bike in a horizontal position, cycling on an exercise bike in a vertical position, ascending stairs, and descending stairs. The reasons for the confusion between similar activities can be summarized in two aspects. Firstly, within the same individual, different activities may have similar activity cycles or amplitudes, which can cause confusion in activity recognition and result in a decrease in overall accuracy.

The kernel Fisher discriminant analysis (KFDA) is a powerful extension of the Fisher discriminant analysis (FDA) [25]. It has been shown to be highly effective in various pattern recognition and classification tasks. While the traditional FDA is primarily designed for linearly separable data, the KFDA extends its capabilities by allowing the analysis of nonlinearly separable data using kernel functions. The KFDA method serves as a robust nonlinear classifier that is suitable for pattern recognition, classification, and regression analysis tasks [26]. It is capable of capturing the nonlinear relationships between the input and output variables in a dataset and demonstrates good generalization performance in various practical problems.

The motivation for this paper is derived from [27,28]. We considered similar issues by utilizing the KFDA method prior to proceeding with data classification. Numerous applications in the field of machine learning have been submitted to the KFDA. To address the issue of confusion between similar activities in single-model human activity recognition and improve the overall accuracy of recognizing multi-class activities, we took inspiration from the successful approaches used by [27] and other researchers in solving similar problems related to lithofacies identification. We decided to utilize KFDA (kernel Fisher discriminant analysis) to preprocess the similar activity data before conducting classification. In this paper, we propose a multi-layer neural network model based on the KFDA. This approach involves preprocessing steps, followed by initial classification using a random forest method. Subsequently, the KFDA is applied to process the data. Finally, SVM is employed for the detailed classification of ambiguous actions. The end result is a robust neural network classification model that effectively tackles the challenge of differentiating between similar activities.

In this paper, we propose the XR-KS (detailed description is given in Section 2) design aimed at addressing the issue of confusion between similar activities. To address the issue of similar activity feature similarity, we propose an SVM classification approach that utilizes KFDA. This approach effectively categorizes similar activities. Additionally, we conducted classification experiments on four common benchmark datasets and performed detailed analyses on these datasets. We compared our model to mainstream classification models. Experimental results demonstrate that our model exhibits excellent classification performance.

The remaining sections of this paper are organized as follows: Section 2 provides a brief introduction to the work carried out in this paper, along with details about the dataset used. Section 3 conducts a basic data analysis and employs appropriate data preprocessing techniques. This section introduces our proposed approach for human motion, which is based on a multi-layer classifier. Section 4 presents the experimental setup, provides results for our proposed method on multiple datasets, and offers an analysis and discussion of these results. Finally, in Section 5, we will summarize the insights gathered from these experiments and outline future directions.

## 2. Modeling Framework and Database

Within human activity recognition (HAR) research, various datasets have been previously published. Notably, the UCI (University of California, Irvine, CA, USA) HAR dataset holds a prominent position due to its extensive usage in numerous studies and comparative analyses [29]. Equally noteworthy is the WISDM (wireless sensor data mining) dataset [30], which has also gained significant recognition. Additionally, datasets like UCI DSA [31] and IM-WSHA [32] have been widely employed in research endeavors. Besides these datasets, there are several others that are not explicitly detailed in this article. The subsequent sections will offer a comparative analysis, presented in Table 1, highlighting the strengths and weaknesses of these four primary datasets.

To highlight these differences, a qualitative comparison between these three datasets is presented in Table 1.

While the data collected from UCI DSA may appear simpler in comparison to UCI HAR, WISDM, and UCI ADL, UCI DSA captures a wide range of 19 different human activities. Unlike other datasets, it better represents complex human activities and serves as a more comprehensive showcase for our model in this paper. We conducted experiments using the four databases, but in the following sections, we focus our narrative on UCI DSA.

The UCI DSA data in this paper were obtained from measurements of human activity by miniature inertial sensors and magnetometers in different parts of the body. Sensor data were collected from a total of 8 subjects performing 19 different activities. The total signal duration for each subject for each activity was 5 min. The sensor unit was calibrated to acquire data at a 25 Hz sampling frequency. The 5-min signal was divided into 5-s segments, resulting in 480 (=60 × 8) signal segments for each activity.

Eight volunteers participated, resulting in a dataset comprising 9120 instances. This dataset intricately describes the data captured from various sensors, measuring activities performed by different subjects within the same time intervals. We consolidated this textual dataset into a CSV file with two columns: subject ID and activity type.

After preprocessing the data and utilizing filtered features, our team developed a practical algorithm to classify 19 human behaviors. Given the vast amount of data and the inherent similarities among human activities, direct classification using a single machine learning algorithm could lead to confusion and decreased accuracy.

**Figure 1:**
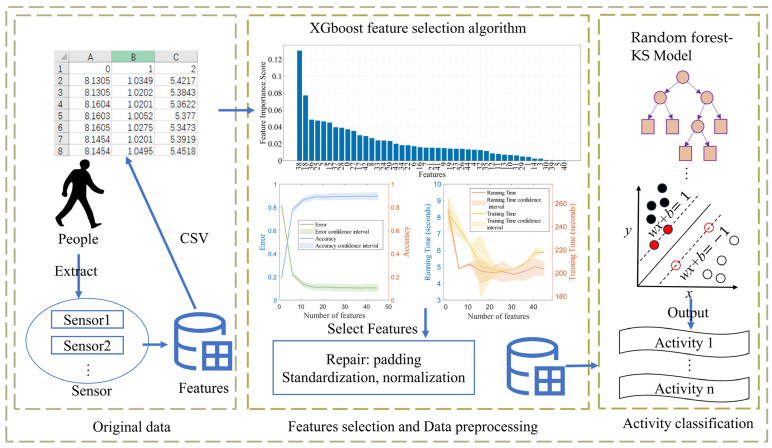
Overview of the system workflow. (The collected human activity data are processed and then classified before utilizing the XR-KS model).

Our approach includes data preprocessing, feature selection, and a two-layer model classification, roughly as shown in Figure 1.

The model proposed in this paper is named XR-KS. First, we define the XR-KS model as follows: The XR-KS model is a two-tiered model. The XR model is a random forest model that utilizes the XGBoost feature selection algorithm. On the other hand, the KS model is a support vector machine model that is based on kernel Fisher discriminant analysis. We will detail these two models below.

The approach in this paper begins by processing data that have been minimized and normalized. Inspired by the success of Zhang et al. in ozone prediction, who utilized the BO-XGBoost-RFE algorithm [33] for feature selection, we decided to employ the advanced XGBoost model for feature selection. The data with the filtered features are then used as the initial dataset, and a random forest classification model is employed for classification, which produces preliminary classification results. This entire process constitutes the first layer of the XR model.

However, we observed that certain similar human behaviors are prone to causing confusion in the classification results. Therefore, we introduce kernel Fisher discriminant analysis to preprocess the data before employing a support vector machine for classification. This iterative process continues until no more instances of confusing activities are encountered. The models derived from these processes are called the KS models.

**Figure 2:**
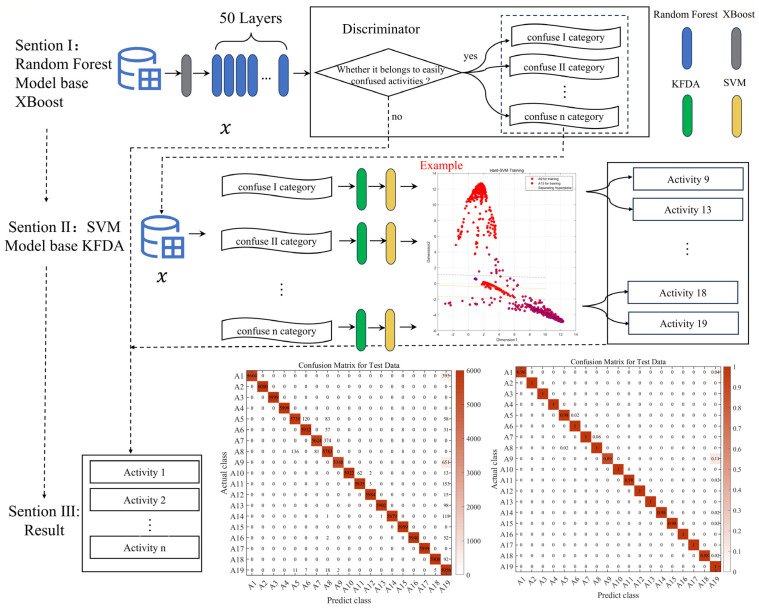
XR-KS model workflow diagram. (The architecture of XR-KS. The first layer of this model relies on a random forest model based on the XGBoost feature selection algorithm, and then, based on the results of the first layer, the second layer uses the support vector machine model based on kernel Fisher discriminant analysis to output the final result, which is very effective in classifying similar human activities).

To provide a more detailed overview of our efforts in addressing similar activities and distinguishing them from other actions in confusing scenarios, we created Figure 2 to illustrate our model.

## 3. Method and Data Preprocessing

### 3.1. Data Preprocessing

#### 3.1.1. Intuitive Data Processing

In this section, the preprocessing work is illustrated using the UCI DSA dataset as an example, in order to avoid unnecessary complexity in the article. We downloaded the dataset from the official UCI website [31] and found it to be somewhat disorganized. To streamline the dataset, we consolidated the original files into a CSV file. Additionally, in order to simplify the lengthy labels in the “Behavior” column of the dataset, we adopted an abbreviated format. Since the actual experiments do not require specific information such as IDs and names, we found it unnecessary to include them. This processing aligns with the original dataset, for example, by replacing “sitting” with “A1”. For detailed information, please refer to Table 2.

#### 3.1.2. Standardization and Normalization

**Figure 3:**
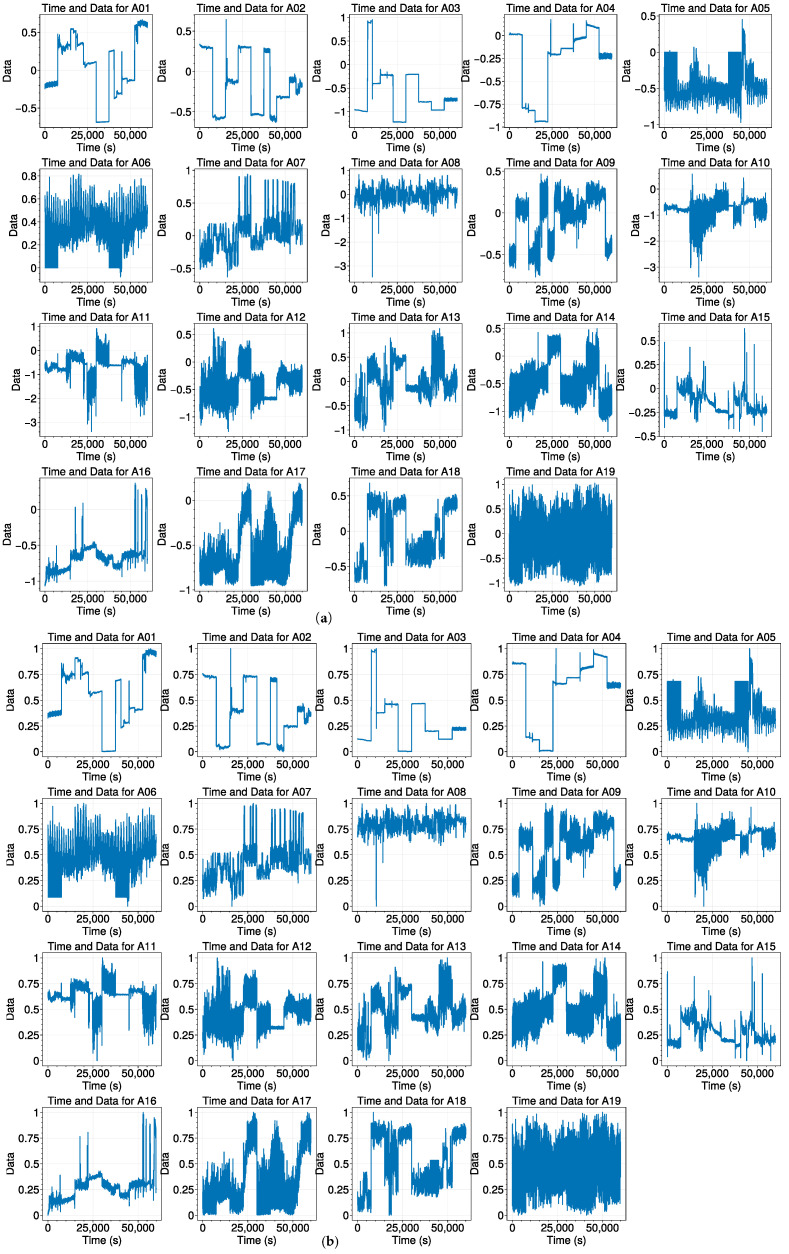
Pre- and post-data preprocessed from A1 to A19. (Minimizing and normalizing aim to scale data consistently, ensuring equal influence from variables in the model). (**a**) Original data; (**b**) data after preprocessing.

To address potential variations in sensor data collection and enhance the classification performance of our proposed model, we conducted a comprehensive examination of data samples. This involved randomly selecting a metric and comparing it with 19 different activities. The initial dataset, illustrated in Figure 3a, comprised 60,000 sample points across various activities for this metric. To ensure consistency and facilitate improved classification, we applied standardization and normalization methods during data preprocessing.

As shown in Figure 3b, after our preprocessing steps, it becomes evident that all data points now fall within the standardized range of 0 to 1. Despite this transformation, the fundamental characteristics of the data are preserved. This meticulous preprocessing not only mitigates potential discrepancies in sensor readings but also enhances the robustness of the classification framework for the diverse set of activities.

Firstly, we propose the XGBoost–random forest model by combining the XGBoost feature selection algorithm with the random forest model. Subsequently, we introduce the XGBoost feature selection algorithm and the random forest model, presenting the combined XGBoost feature selection algorithm–random forest model. Furthermore, we introduce the kernel Fisher discriminant analysis model and SVM model, culminating in the proposal of the KFDA-SVM model by combining these two techniques.

### 3.2. Random Forest Initial Classification Model Based on the XGBoost Feature Selection Algorithm

**Figure 4:**
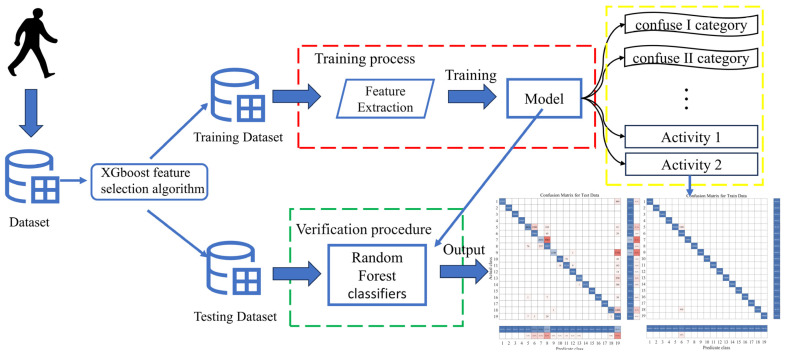
Presentation of the random forest model based on the XGBoost feature selection algorithm workflow.

To achieve a higher initial classification accuracy for subsequent improvements in the second classification stage, it is noted in [34] that utilizing feature selection algorithms can effectively enhance the efficiency of machine learning. Therefore, we initially extract relevant metrics using the XGBoost feature selection algorithm to assess the importance of input metrics. Random forest, an ensemble classifier employing multiple decision trees to train samples and make predictions, is then utilized. In this section, the random forest model based on the XGBoost feature selection algorithm is depicted in Figure 4.

#### 3.2.1. XGBoost Feature Selection Algorithm

XGBoost was first proposed by Chen et al. (2014) [35]. The traditional objective function of GBDT is to predict the target category by stacking the residual trees from different iterations. XGBoost improves upon the traditional GBDT objective function by incorporating a regularization term into the original function and reducing the number of regularization terms. XGBoost improves the traditional GBDT objective function by incorporating a regularization term into the original function. This addition helps to mitigate overfitting and accelerate convergence. XGBoost improves the traditional GBDT objective function by incorporating a regularization term into the original function. This addition helps to mitigate overfitting and enhances the convergence speed.

The objective function of the model is shown in Equation (1) as follows:

(1) $$Obj^{\left(i\right)}=\sum_{i=1}^{n}\left(L\left(y_{i},{\hat{y}}_{i}\right)+\Omega \left(f_{i}\right)\right),$$

where $L(y_{i},{\hat{y}}_{i})$ is the loss function of the squared difference between the true value $y_{i}$ and the predicted value ${\hat{y}}_{i}$. Ω(ϕ) is the regularization term.

(2) $$\Omega (f)=\gamma T+\frac{1}{2}\lambda ∥w{∥}^{2},$$

where γ is the difficulty coefficient of the tabular tree splitting that is used to control the generation of the tree, T denotes the number of leaf nodes, and λ denotes the L2 regularity coefficient.

Taylor’s second-order expansion of the objective function from Equation (1) is as follows:

(3) $$Obj^{\left(i\right)t}=\sum_{i=1}^{n}\left[g_{i}f_{t}\left(x_{i}\right)+\frac{1}{2}h_{i}f_{t}^{2}\left(x_{i}\right)\right]+\Omega \left(f_{i}\right)+C$$

The definition of a tree is as follows:

(4) $$f_{t}(x)=w_{q(x)},\;w\in R^{T},\;q:R^{d}\to \{1,2,\ldots ,T\}$$

where q represents the structure of the tree: Map input samples $x_{i}\in R^{d}$ to leaf nodes; T is the number of leaf nodes; and w is a one-dimensional vector with length T, which represents the weight of the leaf nodes.

The objective function can be rewritten as follows:

(5) $$\begin{array}{ll} Obj^{\left(i\right)} & =\sum_{j=1}^{t}\left[G_{j}w_{i}+\frac{1}{2}\left(H_{j}+\lambda \right)w_{j}^{2}\right]+\gamma T\\ & =-\frac{1}{2}\sum_{j=1}^{T}\frac{{\left(\sum_{i\in I_{j}}g_{i}\right)}^{2}}{\sum_{i\in I_{j}}h_{i}+\lambda }+\gamma T. \end{array}$$

where $I_{j}$ is the sample set of the j-th leaf node, and γ is the weight factor.

**Figure 5:**
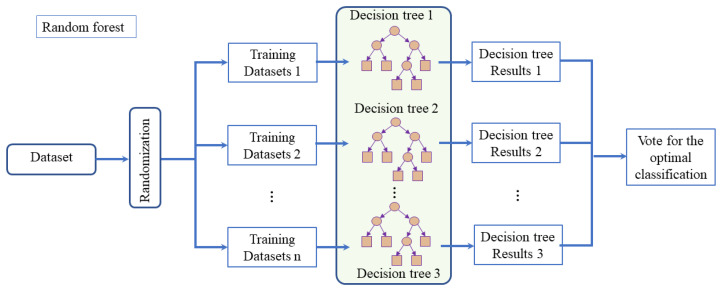
Random forest algorithm flow chart.

The main features that can classify human behavior have been extracted in the previous steps. Considering the relationship between these data and the fact that the training samples are discrete and the data volume is substantial, the random forest algorithm is being considered for network training. The general algorithmic flow of the random forest is illustrated in Figure 5. In order to initially identify multi-class activities, the random forest classification algorithm, which has shown excellent performance in supervised learning, is chosen as the layer 1 classifier.

#### 3.2.2. Random Forest Based on the XGBoost Feature Selection Algorithm

Random forest is a composite classification model composed of many decision tree classification models $\left\{h\left(X,\Theta_{k}\right),k=1,\ldots \right\}$, and the parameter set $\left\{\Theta_{k}\right\}$ is a collection of independently and identically distributed random vectors. Under the given independent variables X, each decision tree classification model selects the optimal classification result through a majority vote. The basic idea is to first use bootstrap sampling to extract k samples from the original training set, with each sample having the same sample size as the original training set. Then, k decision tree models are built for the k samples, resulting in k different classification results. Finally, based on these k classification results, a majority vote is used to determine the final classification result for each record.

The final classification decision in a random forest is made by training through k rounds, obtaining a sequence of classification models $\left\{h_{1}\left(X\right),h_{2}\left(X\right),\cdots ,h_{k}\left(X\right)\right\}$, and using them to create a multi-classification model system. The ultimate classification result of this system is determined using a simple majority voting method:

(6) $$H(x)=\arg\underset{Y}{\max}\sum_{i=1}^{k}I\left(h_{i}(x)=Y\right),$$

where, $H\left(x\right)$ is a multi-classification model, $h_{i}$ is an individual decision tree classification model, and Y represents the output variable.

The specific implementation of the ideas, as demonstrated in Algorithm 1, integrates the XGBoost feature selection algorithm with random forest modeling to develop a unique classification model. The advantage of this lies in its ability to effectively select features from the classification dataset.
**Algorithm 1:** Random forest model based on the XGBoost feature selection algorithm**Input:** Let $D=(x_{1},y_{1}),\ldots ,(x_{N},y_{N})$ denote the training data, with  $x_{i}=(x_{1},\ldots ,x_{k})$  number of trees M>0. **Output:** Prediction of the random forest at $x_{i}$ and random forest model.1: Train the XGBoost model based on the data D.2: **For** a=1,…,k **do**3:   The i metric is scored according to the XGBoost model, and then the scoring data are obtained.4: **End**5: Output m metrics and replace them with metrics from the D dataset.6: Randomly divide D into train data R and test data L in a certain ratio.7: Set the objective function $wx^{T}+b=0$ using train data R and test data L.8: **For** i=1,…,M **do**9:    Find suitable w and b, meet:10:  **For** j=1,⋯,J **do**11:  **If** $y_{j}=+1$ **then**12:  $wx_{j}+b\geq +1$13:  **else if** $y_{j}=-1$ **then**14:  $wx_{j}+b\leq -1$15:  **End**16: **End**17: Adjustment to test set L.18: Obtain the optimal w, b.19: **For** k=1,…,M **do**20:  Train the kth tree based on the test set and the training set to obtain $h_{i}(x)$.21: **End**22: Classification of new samples is based on grown decision trees, utilizing a majority vote mechanism to determine the final classification result.23: $f\left(x_{t}\right)=\;\mathrm{majority}\;\mathrm{vote}\;\left\{h_{i}\left(x\right)\right\}\;(i=1,2,\cdots ,k)$.

### 3.3. Second-Layer SVM Classification Based on Kernel Fisher Discriminant Analysis

**Figure 6:**
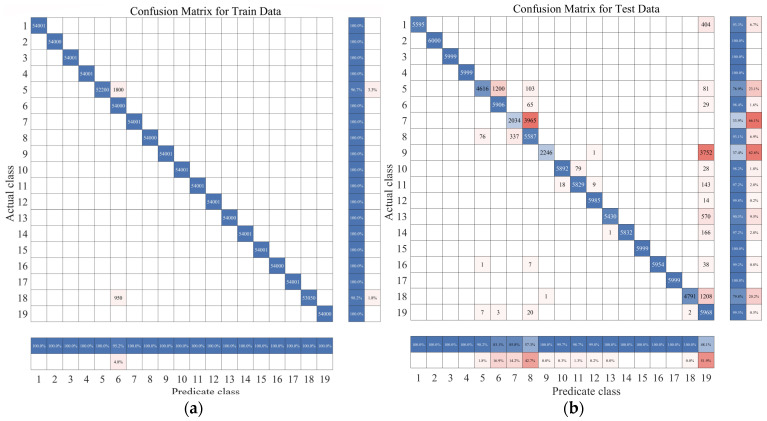
Confusion matrix of test set and training set random forest training. (The experimental setup is like the one in Figure 9). (**a**) Confusion matrix of random forest model by train set; (**b**) confusion matrix of random forest model by test set.

**Figure 7:**
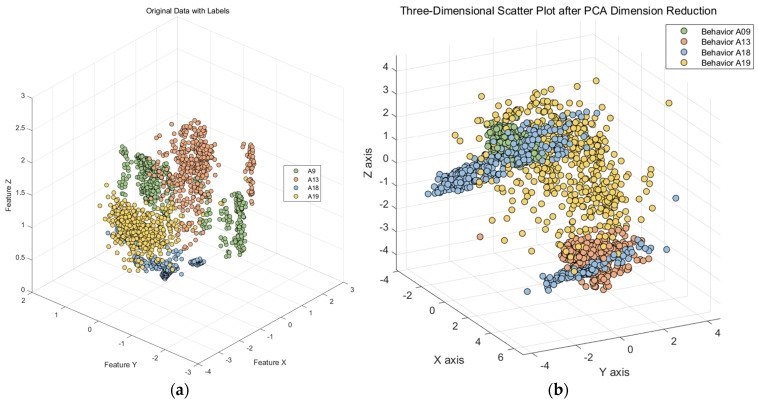
Primary feature and PCA feature. (**a**) Origin data; (**b**) data after PCA.

**Figure 8:**
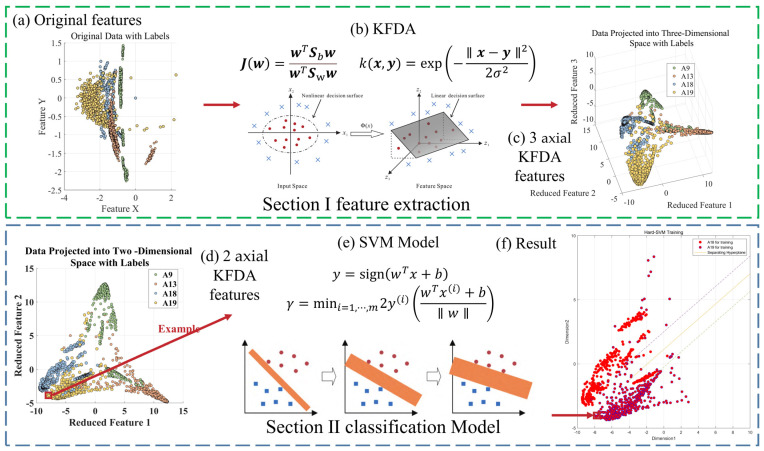
SVM model workflow based on kernel Fisher discriminant analysis. (The second-layer classification model ensures close proximity for (**a**) similar activities. (**b**) After applying KFDA, (**c**) 3 axial data and images are generated. (**d**) The two-dimensional data for the 2 axial image, (**e**) SVM classification, (**f**) the final results).

In Figure 6, we observe challenges in classifying actions such as ascending and descending stairs due to intricate details. Additionally, Figure 7 reveals that PCA and small intra-class distances in the original features hinder effective classification. To mitigate confusion between similar actions, we employ two key steps. First, we employ KFDA (kernel Fisher discriminant analysis) for feature dimensionality reduction to improve the discrimination of similar activities. This aims to increase the separation between distinct actions in the data space, thereby facilitating subsequent SVM classification. The workflow mirrors the one shown in Figure 8.

**Figure 9:**
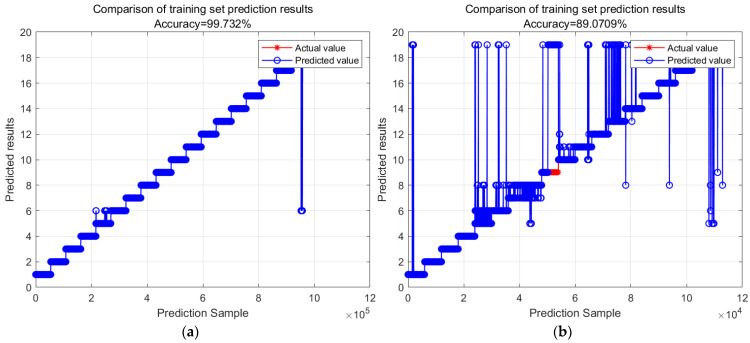
Comparison chart of random forest model recognition results. (The training and test set ratio was set to 9:1, and the experiment was repeated five times). (**a**) Radom forest model by train set; (**b**) random forest model by test set.

#### 3.3.1. Principle of Kernel Fisher Discriminant Analysis

KFDA is a pattern recognition and classification method based on kernel techniques and is an extension of Fisher discriminant analysis. KFDA is designed to handle nonlinearly separable data by mapping the data to a high-dimensional feature space, thereby improving classification performance. We describe KFDA in conjunction with [27]. Kernel Fisher discriminant analysis (KFDA) was first proposed by Schölkopf et al. in 1997 [36] and can be expressed as the maximization Equation (7):

(7) $$J(w)=\frac{w^{T}S_{b}w}{w^{T}S_{w}w},$$

where $S_{w}$ represents the within-class scatter matrix, $S_{b}$ is the between-class scatter matrix, and w denotes the projection vector.

The above problem can be equated to finding the generalized eigenvectors of the eigenvalue problem:

(8) $$S_{b}v_{i}=\lambda_{i}S_{w}v_{i}.$$

where the eigenvalues $\lambda_{i}$ represent the discriminative power of each projection vector. Once we obtain the projected vector v, it can be used for classification instead of the original vectors with a linear classifier.

The limitations of the LDA method are primarily due to its inherent linearity, especially when applied to nonlinear problems [37]. In contrast, the KFDA method, which is an improved version of LDA that uses a kernel trick, overcomes these limitations. KFDA is better suited for the analysis of high-dimensional data and complex systems. It is easy to implement and is characterized by its adaptability and generalizability.

**Figure 10:**
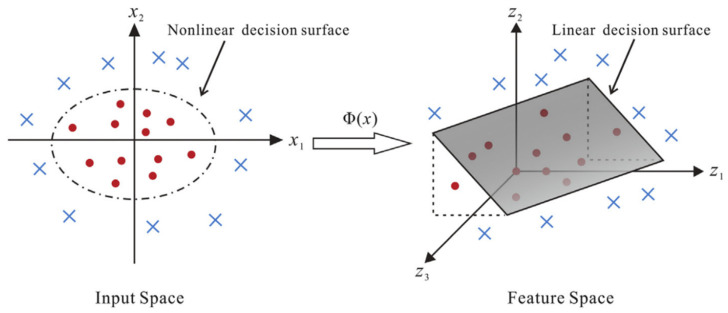
Transformation process illustration of a KFD model. A nonlinear mapping function ϕ(x) converts a nonlinear problem in the original (low-dimensional) input space to a linear problem in a (higher-dimensional) feature space (from [27]).

The core concept of KFDA is to map the original input data using a nonlinear mapping function ϕ into a high-dimensional feature space F, typically a nonlinear space (see Figure 10). Through this transformation, nonlinear relationships within the input data are indirectly transformed into linear relationships. LDA is then applied to extract the most significant discriminating features in this feature space. To overcome the computational challenges of calculating ϕ, researchers add kernel parameters to express functional relationships for nonlinear mappings.

The goal of KFDA is to find a set of projection vectors that maximize the inter-class distance while minimizing the intra-class distance within the feature space. This is achieved by maximizing the kernel Fisher criterion.

(9) $$J(\alpha )=\frac{\alpha^{T}K_{b}\alpha }{\alpha^{T}K_{w}\alpha },$$

where α represents the projection vector, $K_{b}$ represents the kernel between-class scatter matrix, and $K_{w}$ is the kernel within-class scatter matrix in the feature space.

The described information in Equation (9) can be reformulated as solving the generalized feature equation, thus reducing redundancy:

(10) $$K_{b}\alpha =\lambda K_{w}\alpha ,$$

where λ is the nonzero eigenvalue of projection vector α. Let $\alpha_{opt}=(\alpha_{1},\ldots ,\alpha_{M})$ be the optimal projection vector, it is also the maximum eigenvalue from Equation (4). $\lambda_{1},\ldots ,\lambda_{M}$ are the eigenvalue of $\alpha_{1},\ldots ,\alpha_{M}$, respectively, and $\lambda_{1}\geq \cdots \geq \lambda_{M}$. The number of vectors m is calculated using the cumulative contribution rate $\sum_{i=1}^{m}\lambda_{i}/\sum_{i=1}^{M}\lambda_{i}\geq 90\%$.

If $\alpha_{opt}$ is known, the nonlinear decision function f(x) of KFDA is as follows:

(11) $$f(x)=\sum_{i=1}^{n}\alpha_{i}k\left(x_{i},x\right),$$

where $\alpha_{i}$ is the coefficient vector of the i kernel, $x_{i}$ is the i one in all the input samples, and k is the kernel function.

#### 3.3.2. Kernel Parameter Optimization

Among these kernel functions, the Gaussian kernel stands out due to its strong generalization capability and the fact that it requires fewer parameters to be set. This makes it particularly effective in capturing nonlinear relationships. Therefore, in [37], the Gaussian kernel function was chosen as the kernel function and is expressed as shown in Equation (12).

(12) $$k(x,y)=\exp\left(-\frac{∥x-y{∥}^{2}}{2\sigma^{2}}\right),$$

where σ is the width parameter of the Gaussian kernel.

**Figure 11:**
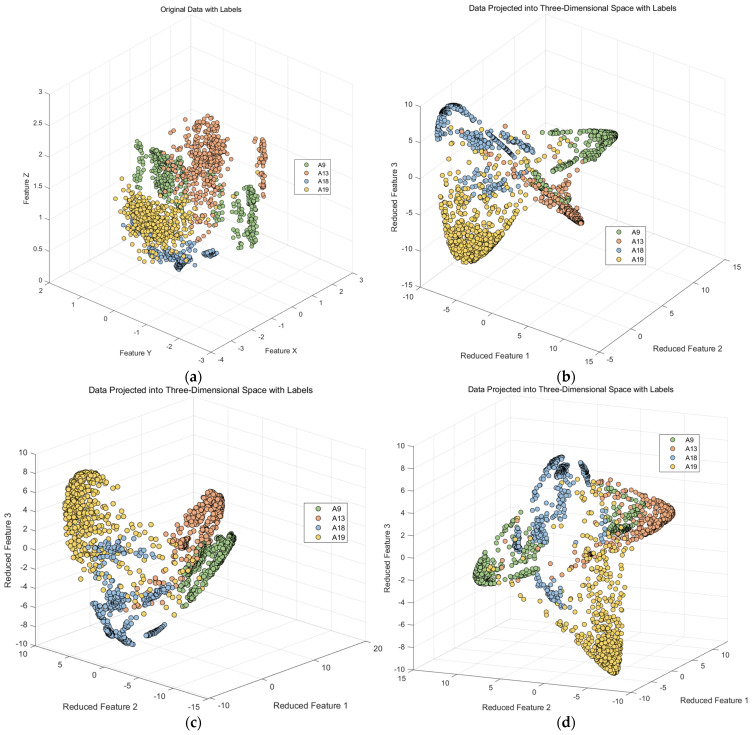

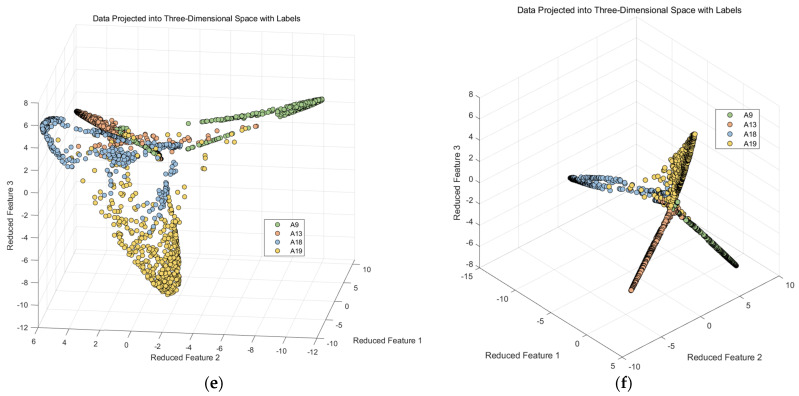
Result of kernel Fisher discriminant analysis with varying parameters. (**a**) σ=N/A; (**b**) σ=1.53; (**c**) σ=0.5; (**d**) σ=1.0; (**e**) σ=2.0; (**f**) σ=5.0.

The kernel parameter σ in the KFDA-SVM model is crucial, influencing the position and distribution of data in the feature space. It significantly impacts the classification efficiency and generalization ability of the SVM model. Figure 11 illustrates the importance of the kernel parameter, showcasing experimental values of 0.5, 1, 2, and 5. The selection of the correct kernel parameter value is a crucial step in attaining optimal results.

#### 3.3.3. SVM Model Based on Kernel Fisher Discriminant Analysis

**Figure 12:**
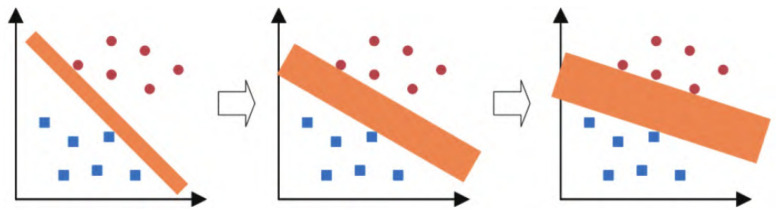
Vector machine classification flow chart. (The figure displays blue and red dots representing distinct classes, with the SVM iteratively seeking the optimal classification hyperplane through continuous refinement).

In order to further categorize the confusion action into specific actions, this paper introduces the support vector machine (SVM) model as a sub-classification model for dividing the confusion action. The principle of the SVM classifier is to find a hyperplane that maximizes the distance between different categories to achieve effective classification. As shown in Figure 12, increasing the width of the classification interval (i.e., maximizing it) reduces the impact of local interference in the training set. Therefore, it can be considered that the last classification method has the best generalization performance and overall applicability. The SVM model can be formulated as follows:

(13) $$y=\mathrm{sign}\left(w^{T}x+b\right),$$

where, x is the feature vector, w is the weight vector, y is the marker vector, and $\mathrm{sign}\left(y\right)$ is the sign function.

When y=1, the sample is positive; when y=−1, the sample is negative, i.e.,

(14) $$\left\{\begin{array}{l} w^{T}x+b>0,y=1,\\ w^{T}x+b\leq 0,y=-1. \end{array}\right.$$

As shown in Figure 12, SVM typically finds the optimal classification hyperplane by maximizing the classification margin. Assuming that the input of the training set consists of a set of x(i) vectors and the output is the set of y(i) vectors, the classification interval is twice the minimum distance from the full set of samples to the hyperplane, which is as follows:

(15) $$\gamma =\underset{i=1,\cdots ,m}{\min}2y^{\left(i\right)}\left(\frac{w^{T}x^{\left(i\right)}+b}{∥w∥}\right).$$

where m is the number of samples. See Algorithm 2.
**Algorithm 2:** SVM model based on kernel Fisher discriminant analysis**Input:** Let $B=\left\{\left(x_{1},y_{1}),\left(x_{2},y_{2}\right),\ldots ,(x_{n},y_{n}\right)\right\}$, kernel value σ. **Output:** Prediction of the SVM at $x_{i}$ and SVM model.1: **For** i=1,…,n **do**2:  Calculate sample size n.3:  Calculate kernel matrix $K_{c}$ = compute_kernel_matrix($X_{c}$, kernel type, degree, σ).4:  Update $Sw+=X_{c}.T\times K_{c}\times X_{c}$.5:  Calculate the inter-class scatter matrix $Sb+=n_{c}\times (mu_{c}-mu)\times (mu_{c}-mu)$.6: **End**7: Construct the projection matrix W, where the columns of W are the selected eigenvectors.8: Return the projection matrix W and data A.9: Set the objective function $wx^{T}+b=0$ using train data $A_{1}$ and test data $A_{2}$.10: **For** i=1,…,M **do**11:    Find suitable w and b, meet:12:    **For** i=1,…,M **do**13:    **If** $y_{j}=+1$ **then**14:    $wx_{j}+b\geq +1$15:    **else if** $y_{j}=-1$ **then**16:    $wx_{j}+b\leq -1$17:    **End**18: **End**19: Obtain the support vector machine model.

**Figure 13:**
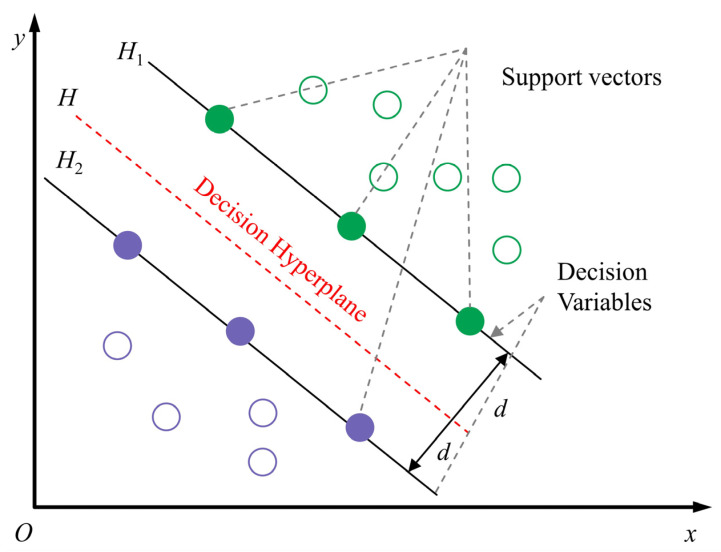
Vector machine classification schematic.(different colors show positive and negative samples. Hollow circles mark support vectors, the points closest to the separating hyperplane. Solid markers represent instances farther away from the hyperplane)..

Mathematically, all sample points that satisfy Equation (15) (i.e., sample points with the smallest Euclidean distance to the classification hyperplane) will be defined as support vectors. Therefore, the set of samples must satisfy the following two cases: if the samples are positive, and if the samples are negative, as shown in Figure 13.

Therefore, the characteristic samples in the sample set should satisfy the discriminant equation when multiplied by the corresponding coefficients.

(16) $$y^{\left(i\right)}\left(w^{T}x^{\left(i\right)}+b\right)\geq 1.$$

## 4. Experimental Setup

### 4.1. Experimental Setting

The experiments were conducted in Guilin, China, on an ASUS computer with the following specifications: an AMD Ryzen 7 4800H processor with Radeon graphics, operating at 2.90 GHz, 16 GB of RAM, and an NVIDIA GeForce GTX 1660 Ti graphics card. The operating system used was Windows 10. We used both MATLAB 2022R and Python 3.9.7 tools to conduct the experiments and validate them on four different datasets: UCI DSA, UCI HAR, WISDM, and UCI ADL. We also conducted a comprehensive evaluation of our approach. To maintain the conciseness of the paper, the following experiments are illustrated using the UCI DSA dataset as an example.

### 4.2. Extraction of Important Features

**Figure 14:**
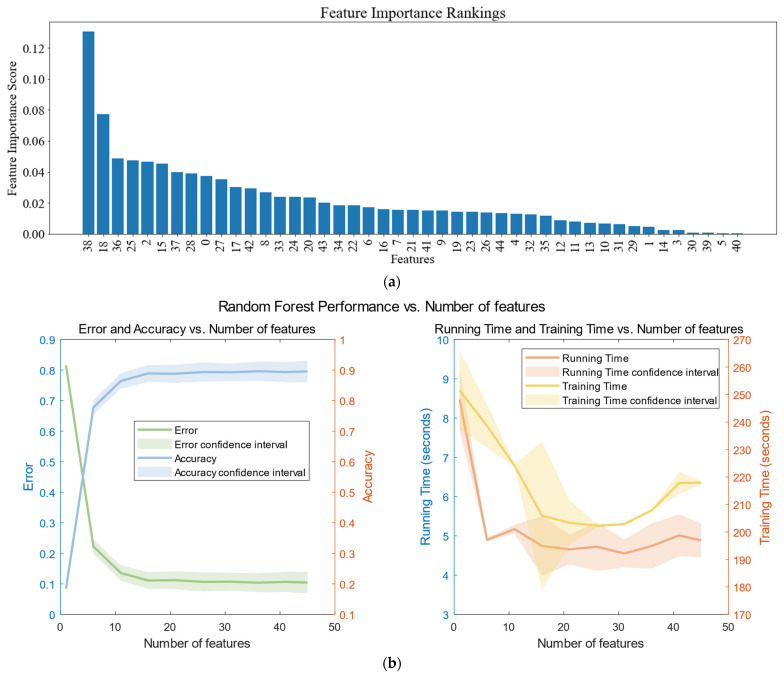
Weights of important features chart and effect of different numbers of features on a random model. (We set the number of features to be from 1 to 46 steps, took 5, and repeated the error experiment for each different n to obtain the mean and confidence interval). (**a**) Result of the XGBoost feature value selection algorithm; (**b**) effect of different numbers of features on a random model.

By utilizing the XGBoost feature value selection algorithm to analyze the 45 features in the dataset, we can evaluate the relative importance of each feature. As shown in Figure 14b, we obtained different experimental results by using the first n features as input to the first layer of the random forest model. These results effectively demonstrate the accuracy and time consumption in various scenarios. Therefore, we selected the first 31 features from Figure 14a to be used in the subsequent multi-layer classifier based on generalized discriminant analysis. To mitigate potential interference with our classification accuracy, we filter out features with lower importance.

The histogram of important feature weights as well as feature selection experiments are shown in Figure 14, and some of the results of the experiments are shown in Table 3.

### 4.3. Extraction of Random Forest Based on the XGBoost Feature Selection Algorithm 

**Figure 15:**
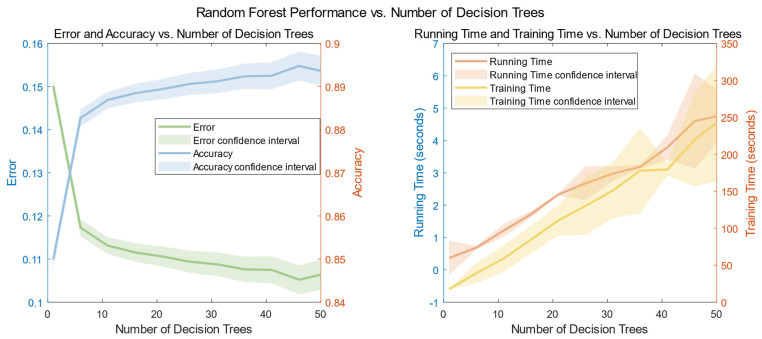
Plot of the number of decisions versus error in the random forest model. (We set the number of decision trees to be from 1 to 50 steps, took 5, and repeated the error experiment for each different n to obtain the mean and confidence interval).

Firstly, using the random forest model in MATLAB, a plot illustrating the relationship between the number of decisions and the error and time was generated, as depicted in Figure 15. Specific results can be found in Table 4.

Examining Figure 9 above, we note that, after a comprehensive evaluation considering computer performance, model accuracy, and overall model reliability, we have concluded that the optimal number of decision trees for this dataset is 50. To thoroughly assess the classification capabilities of the random forest model, a dedicated test dataset was created. Experiments were conducted using MATLAB, involving the uniform partitioning of the entire dataset into different ratios based on various human activities and different volunteers. The outcomes, which detail the influence of these ratios on both training and test set accuracy, are summarized in Table 5.

To better analyze the random forest identification results mentioned above, a confusion matrix plot was created using MATLAB.

We also conducted a random forest classification on data from the other three databases individually, using a similar experimental setup. The experimental results obtained are presented in Table 6.

Observing the four charts above and drawing upon real-world judgment, this study suggests that the main reason for the inconsistency between action recognition results and actual results is the similarity in features among these actions, which makes them easily confused during the algorithmic recognition process. For instance, activities such as climbing stairs, walking, or standing in an elevator demonstrate these similarities. Apart from the mentioned actions, the predictive accuracy for all other actions approaches 100%. This indicates that these actions can be recognized and classified as genuine actions within this layer of the classification model.

The remaining unrecognized actions fall into two main categories. The model classifies A9, A13, and A18 as A19 and confuses A7 and A8 with each other. To facilitate subsequent fine-grained classification models, these similar actions are divided into two main categories, as illustrated in Table 7.

### 4.4. Extraction of SVM Model Based on Kernel Fisher Discriminant Analysis

Taking the four behavior classes (A9, A13, A18, and A19) as an example of Confusing category I, we first extracted three of the most important features from the dataset and created a scatter plot, as shown on the left side of Figure 7. It can be observed that these four behavior classes have a relatively short spatial distribution in these three original features, indicating a small inter-class distance and a large intra-class distance. This does not support activity recognition by the classifier.

Subsequently, we applied a principal component analysis (PCA) for dimensionality reduction, as illustrated on the right side of Figure 7. It represents three randomly selected nonlinear discriminative features extracted from the original features of these four similar activities. In this study, we found that the mapping results of the PCA are not particularly favorable, as the intra-class distance remains small.

Therefore, in this study, we employed kernel Fisher discriminant analysis for dimensionality reduction, focusing on the points that were previously misclassified in the upper layer of a random forest. Kernel Fisher discriminant analysis has a parameter denoted as σ, which can vary. Typically, the range for this parameter is set within [0, 10]. We conducted experiments with different parameter settings and obtained multiple images, as depicted in Figure 11.

**Figure 16:**
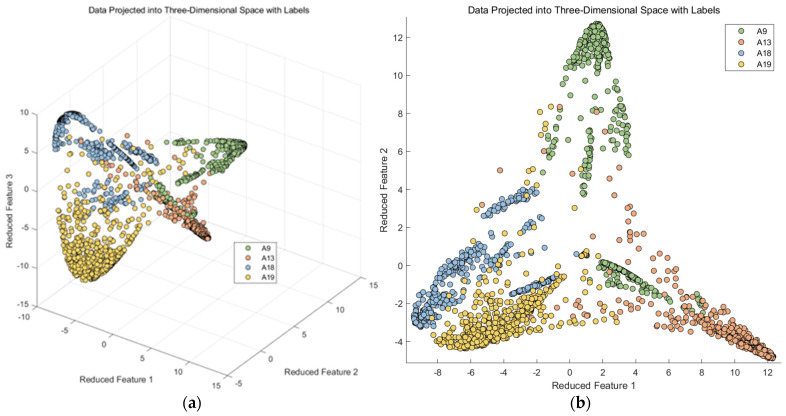
Kernel Fisher discriminant analysis feature. (**a**) 3-axial of data; (**b**) 2-axial of data.

Based on the above experimental results, we can see that in this scenario, upon observing the three-dimensional scatter plot, the data have been categorized into four classes. In order to obtain a clearer visual representation, we selected the two features that performed best in the three-dimensional space and generated a two-dimensional scatter plot, as shown in Figure 16.

**Figure 17:**
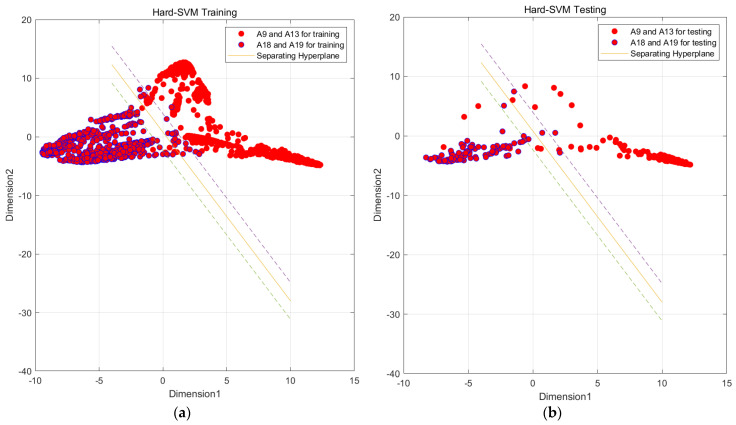
SVM preliminary segmentation A9, A13 and A18, A19 result graph. (Solid line is the main decision boundary, and dashed line is the margin). (**a**) SVM model by train set; (**b**) SVM model by test set.

In this paper, we utilize MATLAB to sub-classify the aforementioned model and input the indicators that have been generalized in the discriminant analysis into SVM as the original data, taking Confusing category I as an example because A9 and A13 are more closely connected and A18 and A19 are also more closely connected. We first subdivide Confusing category I into two large classes, A9, A13 and A18, A19, and then a second subdivision was made to subdivide Confusing category I into more classes, A9, A13, A18, and A19, by a two-layer SVM vector machine. The four classes of activities are A9, A13, A18, and A19, as shown in Figure 17.

**Figure 18:**
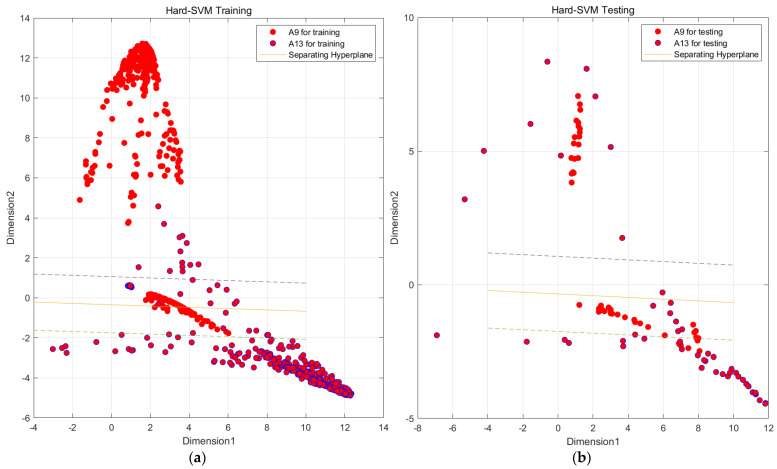
SVM preliminary segmentation A9 and A13 result graph. (Solid line is the main decision boundary, and dashed line is the margin). (**a**) SVM model by train set; (**b**) SVM model by test set.

**Figure 19:**
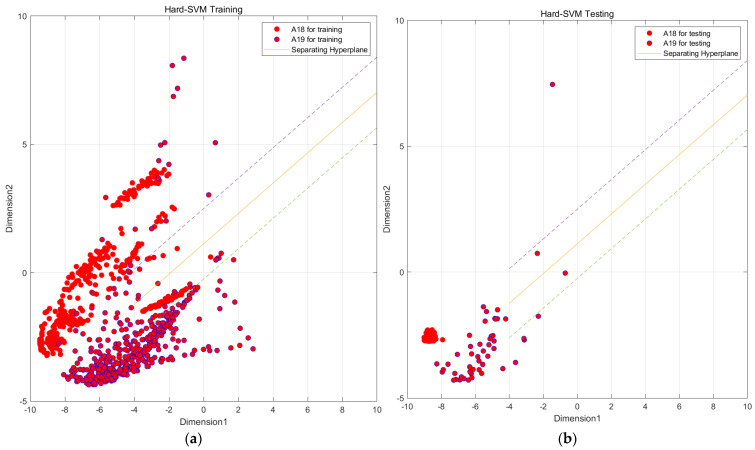
SVM preliminary segmentation A18 and A19 result graph. (Solid line is the main decision boundary, and dashed line is the margin). (**a**) SVM model by train set; (**b**) SVM model by test set.

Through the above steps, the data from Confusing category I were classified into two major classes, A8, A13 and A18, A19, using SVM. In order to achieve a more precise classification, this paper further conducts a fine classification of these two major classes into specific activity classes, as shown in Figure 17, Figure 18 and Figure 19.

Through the steps related to the figure, we are able to classify all the data in Confusion category I into specific active classes using the SVM vector machine meticulous classification. Although the effect of the SVM vector machine fine classification A9 and A13 is not significant, as shown in Figure 19, it is still much better than the initial random forest classification effect. Similarly, we conducted various experiments, as shown in Table 8.

**Figure 20:**
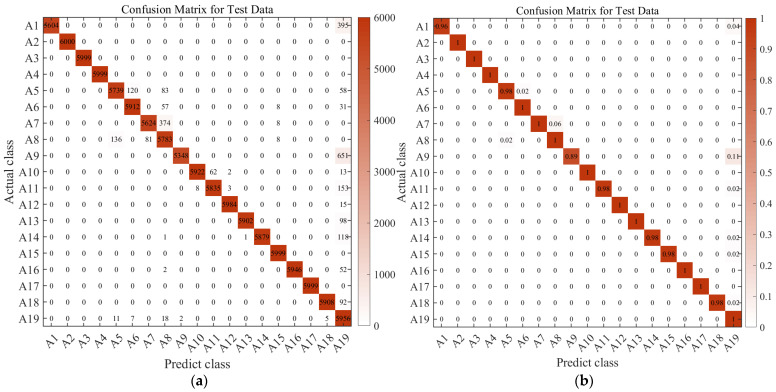
Confusion matrix diagram for the two-layer classifier model XR-KS. (**a**) Test set confusion matrix obtained after correction; (**b**) test set confusion matrix obtained after correction (%).

In this study, a similar operation was performed on Confusing category I, which served as input to the second layer of the support vector machine. This process yielded recognition probabilities for four similar activities of human behavior. The final recognition results for these four activities were determined through a weighted average, which considered the recognition probabilities from the first-layer classifier. Figure 20 illustrates the confusion matrix, revealing substantial improvement in the original actions prone to confusion. The overall accuracy rate increased significantly, rising from 89.07% to an impressive 97.69%.

We also compared our approach with those of others on three datasets: UCI HAR, WISDM, and IM-WSHA, as shown in Table 9.

### 4.5. Extraction of XR-KS Model Generalization Test

**Figure 21:**
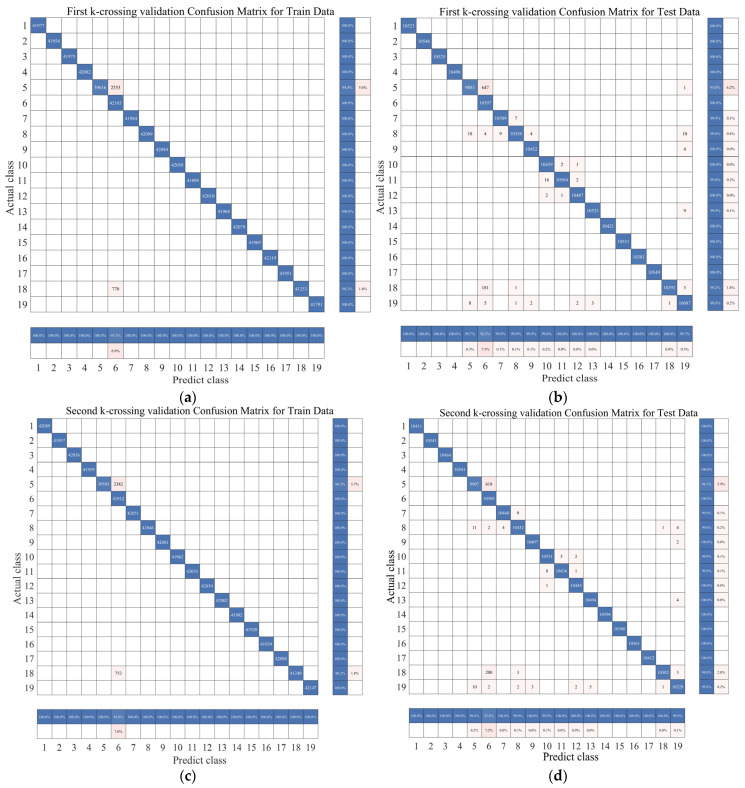

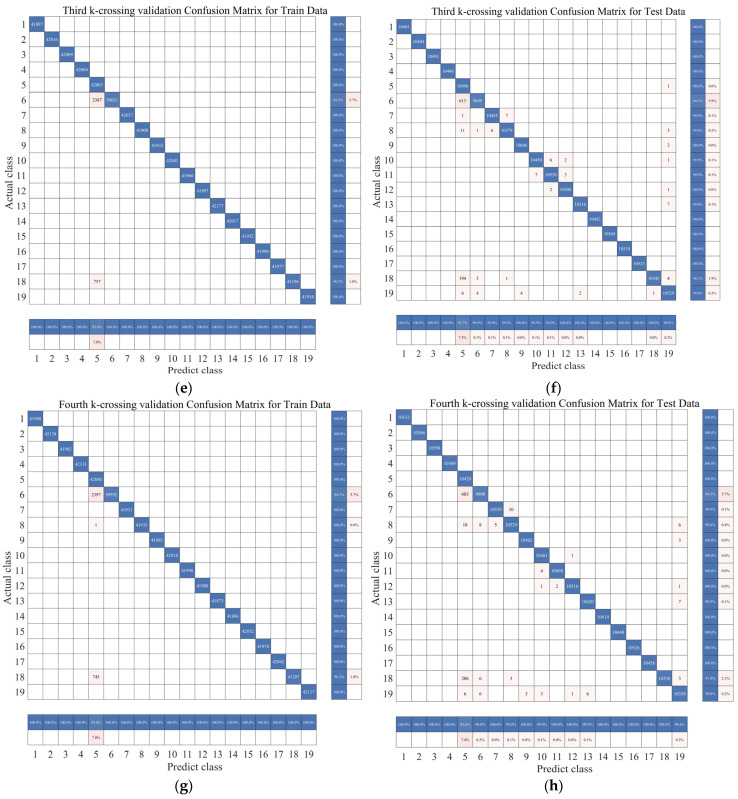

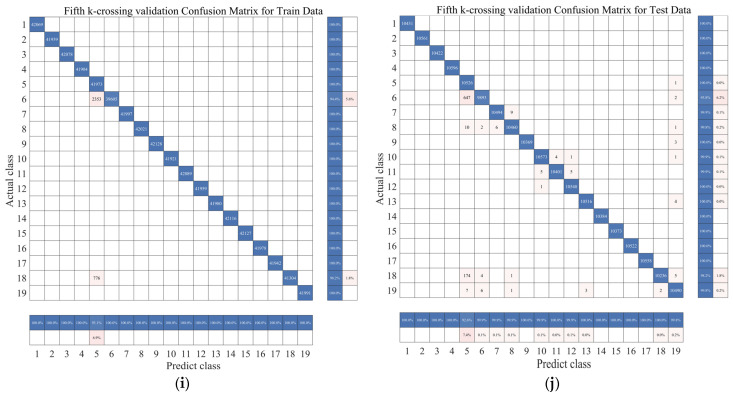
Cross-validation and k = 5 results. (**a**) First-split training set confusion matrix; (**b**) first-split testing set confusion matrix; (**c**) second-split training set confusion matrix; (**d**) second-split testing set confusion matrix; (**e**) third-split training set confusion matrix; (**f**) third-split testing set confusion matrix; (**g**) fourth-split training set confusion matrix; (**h**) fourth-split testing set confusion matrix; (**i**) fifth-split training set confusion matrix; (**j**) fifth-split testing set confusion matrix.

Through research [50], it is known that testing the generalizability of a model can be performed using K-fold cross-validation. Below, we validate the model. First, we conducted experiments using K-fold cross-validation with the random forest model. In this study, we set K = 5 for validation. The experimental results are shown in Figure 21 and Table 10, demonstrating the strong generalization capability of our model.

**Figure 22:**
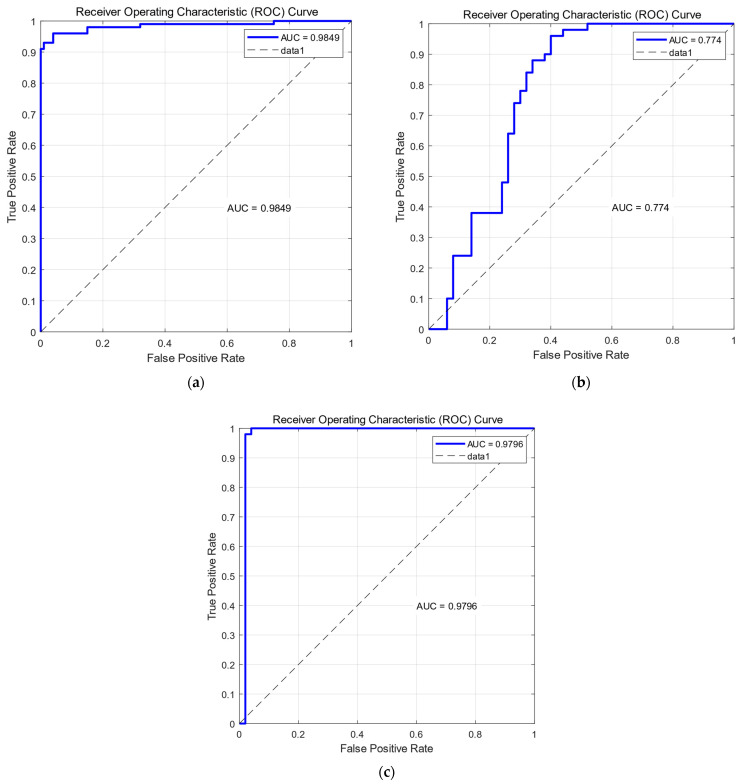
UCI DSA of the SVM model ROC curve and AUC value by train:test = 9:1. (**a**) ROC curves and AUC values of SVM confounded A9, A13 and A18, A19 class result plots; (**b**) ROC curves and AUC values of SVM confounded A9 and A13 class result plots; (**c**) ROC curves and AUC values of SVM confounded A18 and A19 class result plots.

Through the study conducted by Fawcett [51] on ROC curves, we aim to assess the effectiveness of SVM by analyzing the ROC values. The results obtained from the model with a training-to-testing ratio of 9:1 are presented in Figure 22, while the results of other models with varying ratios can be found in Table 11. The above results show that our model is well trained.

## 5. Conclusions

This study proposes an approach to identifying similar activities by introducing a multi-layer model called XR-KS. The approach involves filtering all the features of the data using the XGBoost feature selection algorithm and selecting a subset of features for random forest classification. This method aims to improve the efficiency of classification. Based on the classification results mentioned above, we filtered out the data points that were difficult to identify. We then utilized the SVM model with kernel Fisher discriminant analysis to identify these points. Firstly, we applied the kernel Fisher discriminant analysis to distinguish similar activities based on the data. Next, we used the SVM model to classify the data, resulting in a satisfactory recognition outcome. Additionally, we employed k-fold cross-validation and ROC curves to validate our model separately. The results confirmed that our model exhibits strong generalization capabilities. Our method can identify similar human activities very well. However, we also observed that the recognition effect of A9 and A13 is not very good. Therefore, we will investigate a more suitable model and its simulation algorithm to achieve higher accuracy in recognizing human activity in A9 or A13. This research, however, is subject to several limitations.

- The differentiation of similar activities in the first and second tiers of this thesis is based on subjective perception and data.

Our future research work can be focused on the following three points:

- Extend the proposed technique to handle classification tasks involving similar activity data, such as typing and handwriting. Extend the proposed technique to handle generative tasks in challenging driving conditions, such as datasets with limited features and insufficient data samples.
- Enable the models in the article to automatically distinguish between similar human activities and automate the process of discrimination is a future research direction.
- Starting with data collection, it is important to design and implement a robust and targeted sensor data collection scheme and algorithm. For instance, we should consider removing noise during data collection and focusing on collecting data that facilitate the identification of human activity.

## Figures and Tables

**Table 1:** Comparison between datasets: UCI DSA, UCI HAR, WISDM, and UCI ADL.

	UCI DSA	UCI HAR	WISDM	IM-WSHA
Type of activity studied	Short-time	Short-time	Short-time	Short-time
Different volunteers	Yes	Yes	Yes	Yes
Volunteer numbers	4	30	36	10
Fixed sensor frequency	Yes	Yes	Yes	Yes
Instances	1,140,000	10,299	1,098,207	125,955
Sensors	Acc. and gyro.	Acc. and gyro.	Acc	Acc. and gyro.
Sensor data collection	Comprehensively	Comprehensively	Selectively	Selectively
Sensor type	Phone	Phone	Phone	IMU
Sensor number	3	3	1	3
Activities type	19	6	6	11

**Table 2:** Table of specific actions and corresponding codes in the text.

Behavior	Codes	Behavior	Codes
Sitting	A1	walking on a treadmill with a speed of 4 km/h (15 deg inclined positions)	A11
Standing	A2	running on a treadmill with a speed of 8 km/h	A12
Lying on back	A3	exercising on a stepper	A13
Lying on right side	A4	exercising on a cross trainer	A14
Ascending stairs	A5	cycling on an exercise bike in horizontal	A15
Descending stairs	A6	cycling on an exercise bike in vertical positions	A16
Standing in an elevator still	A7	rowing	A17
Moving around in an elevator	A8	jumping	A18
Walking in a parking lot	A9	playing basketball	A19
Walking on a treadmill with a speed of 4 km/h (in flat)	A10		

**Table 3:** Number of features and error rate, accuracy rate, training times, and running time (mean ± std).

Number of Features	Error Rate	Accuracy Rate	Training Time (s)	Running Time (s)
1	81.5438 (±1.13)%	18.4561 (±1.13)%	251.53 (±7.292)	8.1802 (±0.165)
6	22.1995 (±1.24)%	77.8004 (±1.24)%	238.55 (±4.063)	4.9149 (±0.08)
21	11.1931 (±1.49)%	88.8068 (±1.49)%	203.36 (±4.209)	4.5172 (±0.78)
31	10.7098 (±1.62)%	89.2901 (±1.62)%	202.83 (±0.151)	4.4230 (±0.73)
41	10.6396 (±1.79)%	89.3603 (±1.79)%	217.82 (±2.107)	4.8204 (±0.68)
45	10.4229 (±1.86)%	89.5770 (±1.86)%	217.89 (±0.278)	4.7276 (±0.89)

**Table 4:** Number of decisions and error rate, accuracy rate, training times, and running time (mean ± std).

Number of Decisions Tree	Error Rate	Accuracy Rate	Training Time (s)	Running Time (s)
1	15.0119 (±1.12)%	84.9881 (±1.12)%	13.3845 (±1.507)	0.2793 (±0.126)
6	11.7283 (±1.25)%	88.2717 (±1.25)%	32.5361 (±0.580)	0.7068 (±0.062)
11	11.3098 (±1.39)%	88.6902 (±1.39)%	53.4840 (±0.665)	1.1963 (±0.019)
21	11.0589 (±1.51)%	88.9411 (±1.51)%	95.7831 (±0.345)	2.2481 (±0.059)
31	10.8791 (±1.46)%	89.1209 (±1.46)%	141.0272 (±4.273)	3.2747 (±0.017)
41	10.7501 (±1.81)%	89.2499 (±1.81)%	187.3081 (±10.173)	4.4448 (±0.106)
50	10.5211 (±1.95)%	89.4789 (±1.95)%	215.6206 (±20.992)	5.0635 (±1.308)

**Table 5:** Random forest model based on XGBoost feature selection algorithm result table of UCI DSA (mean ± std). (The experiments were set up with different ratios of test and validation sets and repeated five times).

Ratio (Training: Testing)	UCI DSA Accuracy Rate and Time		
	Training Data	Testing Data	Running Time (s)
9:1	99.7322 (±0.08)%	89.0709 (±1.59)%	5.124 (±0.29)
8:2	99.5669 (±0.12)%	85.1812 (±1.63)%	5.243 (±0.28)
7:3	99.3541 (±0.13)%	79.3321 (±1.76)%	5.165 (±0.36)
6:4	99.2145 (±0.15)%	74.0732 (±1.86)%	5.146 (±0.15)
5:5	99.6656 (±0.17)%	69.5766 (±2.12)%	5.234 (±0.34)
4:6	99.6706 (±0.21)%	68.6753 (±2.65)%	5.091 (±0.39)

**Table 6:** Random forest model based on the XGBoost feature selection algorithm result table between datasets: UCI DSA, UCI HAR, WISDM, and UCI ADL (mean ± std) (the experimental setup is like the one in Table 5).

Ratio (Training: Testing)	UCI HAR Accuracy Rate and Time			WISDM Accuracy Rate and Time			IM-WSHA Accuracy Rate and Time		
	Training Data	Testing Data	Running Time (s)	Training Data	Testing Data	Running Time (s)	Training Data	Testing Data	Running Time (s)
9:1	100 (±0.01)%	92.7863 (±1.01)%	0.38951 (±0.01)	99.9999 (±0.54)%	92.5161 (±0.58)%	27.7856 (±1.12)	99.9974 (±0.05)%	84.1109 (±1.07)%	4.2978 (±0.51)
8:2	100 (±0.01)%	90.9886 (±1.13)%	0.38611 (±0.02)	99.9018 (±0.76)%	92.4081 (±0.69)%	27.6143 (±1.16)	99.6043 (±0.13)%	81.4639 (±1.13)%	4.3386 (±0.55)
7:3	100 (±0.01)%	91.5774 (±1.22)%	0.38302 (±0.04)	99.8996 (±0.81)%	91.9205 (±0.91)%	27.5283 (±1.08)	99.6632 (±0.14)%	76.5923 (±1.24)%	4.2999 (±0.55)
6:4	100 (±0.01)%	90.6069 (±1.34)%	0.39912 (±0.02)	99.9007 (±0.96)%	91.7434 (±1.21)%	27.1654 (±1.16)	99.6756 (±0.12)%	71.1226 (±1.45)%	4.2382 (±0.61)
5:5	100 (±0.01)%	90.9319 (±1.62)%	0.39054 (±0.03)	99.6061 (±1.15)%	91.7573 (±1.36)%	26.5401 (±1.19)	99.6656 (±0.11)%	68.6637 (±1.32)%	3.9513 (±0.62)
4:6	100 (±0.01)%	88.6398 (±1.72)%	0.37715 (±0.01)	99.9014 (±1.53)%	90.9253 (±1.49)%	25.2154 (±1.18)	99.6706 (±0.18)%	65.8418 (±1.23)%	3.9411 (±0.36)

**Table 7:** Confusing action classification table.

Confussing Category	Easily Confused Actions
Confusing category I	A9, A13, A18, A19
Confusing category II	A5, A6
Confusing category III	A7, A8

**Table 8:** Random forest result (mean ± std) table between datasets: UCI DSA, UCI HAR, WISDM, and UCI ADL.

Ratio (Training: Testing)	A9, A13 and A18,A19 Accuracy Rate and Running Time			A9 and A13 Accuracy Rate and Running Time			A18 and A19 Accuracy Rate and Running Time		
	Training Data	Testing Data	Running Time (s)	Training Data	Testing Data	Running Time (s)	Training Data	Testing Data	Running Time (s)
9:1	97.9444 (±0.13)%	95.0 (±0.16)%	0.9401 (±0.15)	84.0 (±0.15)%	59.0 (±0.16)%	0.1802 (±0.071)	86.4444 (±0.14)%	92.0 (±0.16)%	0.1605 (±0.017)
8:2	98.0 (±0.11)%	96.2512 (±0.13)%	0.8749 (±0.13)	79.8714 (±0.19)%	70.0 (±0.19)%	0.1524 (±0.045)	84.8751 (±0.18)%	95.50 (±0.14)%	0.1413 (±0.065)
7:3	98.0 (±0.15)%	97.0 (±0.15)%	0.4801 (±0.14)	76.0 (±0.16)%	79.6667 (±0.13)%	0.0953 (±0.065)	83.7143 (±0.16)%	94.6667 (±0.16)%	0.09714 (±0.094)
6:4	97.8333 (±0.14)%	97.75 (±0.14)%	0.4146 (±0.16)	73.0 (±0.16)%	85.0 (±0.12)%	0.07241 (±0.014)	83.8333 (±0.16)%	92.0 (±0.17)%	0.07048 (±0.053)
5:5	99.90 (±0.14)%	96.95 (±0.17)%	0.4521 (±0.091)	65.80 (±0.14)%	87.60 (±0.11)%	0.05512 (±0.069)	93.40 (±0.16)%	83.40 (±0.17)%	0.05041 (±0.047)
4:6	99.91 (±0.14)%	97.41 (±0.14)%	0.2413 (±0.075)	84.75 (±0.14)%	85.0 (±0.17)%	0.03338 (±0.064)	92.75 (±0.19)%	85.3333 (±0.16)%	0.02581 (±0.034)

**Table 9:** Comparison of recognition accuracy of the proposed method with other state-of-the-art methods over UCI DSA, UCI HAR, WISDM, and IM-WSHA datasets (Bolding in the table indicates the methodology of this paper)..

Reference Study	Model	UCI DSA	UCI HAR	WISDM	IM-WSHA
Qiu et al. (2016) [37]	Estimation algorithm [38]				80.49%
Gochoo et al. (2021) [38]	RPLB [39]				83.18%
Halim et al. (2022) [39]	hybrid descriptors and random forest [40]				91.45%
Ghadi et al. (2022) [40]	MS-DLD [41]				95.0%
Koşar et al. (2023) [41]	Deep CNN-LSTM [42]		93.11%		
Kobayashi et al. (2023) [42]	MarNASNet [43]		94.50%	88.92%	
Wang et al. (2023) [43]	DMEFAM [44]		96%	97.9%	
Dua, N et al. (2023) [44]	Deep CNN-GRU [45]		96.20%	97.21%	
Imran et al. (2023) [45]	EdgeHARNet [46]			94.036%	
Zhang et al. (2023) [46]	BLSTM [47]		98.37%	99.01%	
Thakur et al. (2023) [47]	CAEL-HAR [48]		96.45%	98.57%	
Li et al. (2016) [48]	MVTS [49]	91.35%			
**Present paper**	**XR-** **KS**	**97.69%**	**97.92%**	**98.12%**	**90.6%**

**Table 10:** Mean (±std) k = 5 and cross-validation XR model results of UCI DSA. (Five repetitions of the experiment were conducted to obtain the mean and confidence intervals).

K	Training Data Accuracy	Testing Data Accuracy	Training Running Time (s)	Test Running Time (s)
1	99.7414% (±0.91)	99.1421% (±0.94)	463.0862 (±8.53)	51.0272 (±1.81)
2	99.8561% (±0.84)	99.1421% (±0.45)	408.6202 (±8.45)	48.6971 (±2.31)
3	99.7651% (±0.57)	99.1421% (±0.41)	370.8408 (±9.54)	42.7105 (±2.45)
4	99.8234% (±0.61)	99.1421% (±0.61)	377.3214 (±7.14)	40.2905 (±2.16)
5	99.8317% (±0.74)	99.1421% (±0.37)	386.5293 (±7.41)	39.6749 (±1.97)

**Table 11:** KS model AUC mean (±std) results of UCI DSA. (Five repetitions of the experiment were conducted to obtain the mean and confidence intervals).

Ratio (Training: Testing)	A9, A13 and A18, A19 AUC	A9, A13 AUC	A18, A19 AUC
9:1	0.98491 (±0.01)	0.77412 (±0.01)	0.97961 (±0.02)
8:2	0.99155 (±0.01)	0.90591 (±0.01)	0.97573 (±0.01)
7:3	0.99229 (±0.01)	0.95818 (±0.01)	0.97361 (±0.04)
6:4	0.99386 (±0.03)	0.97513 (±0.01)	0.96473 (±0.03)
5:5	0.99153 (±0.01)	0.98271 (±0.02)	0.86586 (±0.01)
4:6	0.99324 (±0.01)	0.93727 (±0.03)	0.87724 (±0.01)

## Data Availability

Data are contained within the article.

## References

[1] Vrigkas M., Nikou C., Kakadiaris I. (2015). A review of human activity recognition methods. Front. Robot. AI.

[2] Beddiar D.R., Nini B., Sabokrou M., Hadid A. (2020). Vision-based human activity recognition: A survey. Multimed. Tools Appl..

[3] Hannan A., Shafiq M.Z., Hussain F., Pires I.M. (2021). A portable smart fitness suite for real-time exercise monitoring and posture correction. Sensors.

[4] Wang D., Chen J., Zhao D., Dai F., Zheng C., Wu X. (2017). Monitoring workers’ attention and vigilance in construction activities through a wireless and wearable electroencephalography system. Autom. Constr..

[5] Xu F., Xu F., Xie J., Pun C.M., Lu H., Gao H. (2021). Action recognition framework in traffic scene for autonomous driving system. IEEE Trans. Intell. Transp. Syst..

[6] Zhang H., Xiao Z., Wang J., Li F., Szczerbicki E. (2019). A novel IoT-perceptive human activity recognition (HAR) approach using multihead convolutional attention. IEEE Internet Things J..

[7] Khan M.A., Sharif M., Akram T., Raza M., Saba T., Rehman A. (2020). Hand-crafted and deep convolutional neural network features fusion and selection strategy: An application to intelligent human action recognition. Appl. Soft Comput..

[8] Mekruksavanich S., Jitpattanakul A. (2021). LSTM networks using smartphone data for sensor-based human activity recognition in smart homes. Sensors.

[9] Xiao Z., Xu X., Xing H., Song F., Wang X., Zhao B. (2021). A federated learning system with enhanced feature extraction for human activity recognition. Knowl. Based Syst..

[10] Li H., Shrestha A., Heidari H., Le Kernec J., Fioranelli F. (2019). Bi-LSTM network for multimodal continuous human activity recognition and fall detection. IEEE Sens. J..

[11] Yang C.L., Hsu S.C., Hsu Y.W., Kang Y.C. (2023). HAR-time: Human action recognition with time factor analysis on worker operating time. Int. J. Comput. Integr. Manuf..

[12] Zheng X., Meiqing W., Joaquín O. (2018). Comparison of data preprocessing approaches for applying deep learning to human activity recognition in the context of industry 4.0. Sensors.

[13] Lima W.S., Souto E., El-Khatib K., Jalali R., Gama J. (2019). Human Activity Recognition Using Inertial Sensors in a Smartphone: An Overview. Sensors.

[14] Park S., Feng H., Park C., Lee Y.K., Jung S., Kim J. (2023). EQuaTE: Efficient Quantum Train Engine for Run-Time Dynamic Analysis and Visual Feedback in Autonomous Driving. IEEE Internet Comput..

[15] Gao G., Li Z., Huan Z., Chen Y., Liang J., Zhou B., Dong C. (2021). Human behavior recognition model based on feature and classifier selection. Sensors.

[16] Chen Y., Shen C. (2017). Performance Analysis of Smartphone-Sensor Behavior for Human Activity Recognition. IEEE Access.

[17] Demrozi F., Pravadelli G., Bihorac A., Rashidi P. (2020). Human Activity Recognition Using Inertial, Physiological and Environmental Sensors: A Comprehensive Survey. IEEE Access.

[18] Xia C., Sugiura Y. (2021). Optimizing Sensor Position with Virtual Sensors in Human Activity Recognition System Design. Sensors.

[19] Foerster F., Smeja M., Fahrenberg J. (1999). Detection of posture and motion by accelerometry: A validation study in ambulatory monitoring. Comput. Hum. Behav..

[20] Bouten C., Koekkoek K., Verduin M., Kodde R., Janssen D. (1997). A triaxial accelerometer and portable data processing unit for the assessment of daily physical activity. IEEE Trans. Biomed. Eng..

[21] Bao L., Intille S.S. (2004). Activity recognition from user-annotated acceleration data. International Conference on Pervasive Computing.

[22] Lara O.D., Pérez A.J., Labrador M.A., Posada J.D. (2012). Centinela: A human activity recognition system based on acceleration and vital sign data. Pervasive Mob. Comput..

[23] Jansi R., Amutha R. (2018). A novel chaotic map based compressive classification scheme for human activity recognition using a tri-axial accelerometer. Multimed. Tools Appl..

[24] Vanrell S.R., Milone D.H., Rufiner H.L. (2017). Assessment of homomorphic analysis for human activity recognition from acceleration signals. IEEE J. Biomed. Health Inform..

[25] Billings S.A., Lee K.L. (2002). Nonlinear Fisher discriminant analysis using a minimum squared error cost function and the orthogonal least squares algorithm. Neural Netw..

[26] Mika S., Ratsch G., Weston J., Scholkopf B., Mullers K.R. Fisher discriminant analysis with kernels. Proceedings of the 1999 IEEE Signal Processing Society Workshop.

[27] Dong S., Zeng L., Du X., He J., Sun F. (2022). Lithofacies identification in carbonate reservoirs by multiple kernel Fisher discriminant analysis using conventional well logs: A case study in A oilfield, Zagros Basin, Iraq. J. Pet. Sci. Eng..

[28] Liu Q., Lu H., Ma S. (2004). Improving kernel Fisher discriminant analysis for face recognition. IEEE Trans. Circuits Syst. Video Technol..

[29] Reyes-Ortiz J., Anguita D., Ghio A., Oneto L., Parra X. (2012). Human Activity Recognition Using Smartphones. UCI Machine Learning Repository. http://archive.ics.uci.edu/dataset/240/human+activity+recognition+using+smartphones.

[30] Kwapisz J.R., Weiss G.M., Moore S.A. Activity Recognition using Cell Phone Accelerometers. Proceedings of the Fourth International Workshop on Knowledge Discovery from Sensor Data (at KDD-10).

[31] Barshan B., Altun K. Daily and Sports Activities; UCI Machine Learning Repository: 2013. http://archive.ics.uci.edu/dataset/256/daily+and+sports+activities.

[32] Tahir S.B.U.D., Jalal A., Kim K. (2020). Wearable Inertial Sensors for Daily Activity Analysis Based on Adam Optimization and the Maximum Entropy Markov Model. Entropy.

[33] Zhang B., Zhang Y., Jiang X. (2022). Feature selection for global tropospheric ozone prediction based on the BO-XGBoost-RFE algorithm. Sci. Rep..

[34] Li J., Cheng K., Wang S., Morstatter F., Trevino R.P., Tang J., Liu H. (2017). Feature selection: A data perspective. ACM Comput. Surv. CSUR.

[35] Chen T., Guestrin C. Xgboost: A scalable tree boosting system. Proceedings of the 22nd Acm Sigkdd International Conference on Knowledge Discovery and Data Mining ACM.

[36] Schölkopf B., Smola A., Müller K.R. (1998). Nonlinear component analysis as a kernel eigenvalue problem. Neural Comput..

[37] Schölkopf B., Smola A. (2002). Learning with Kernels: Support Vector Machines, Regularization, Optimization, and Beyond. Massachusetts Institute of Technology.

[38] Qiu S., Wang Z., Zhao H., Hu H. (2016). Using distributed wearable sensors to measure and evaluate human lower limb motions. IEEE Trans. Instrum. Meas..

[39] Gochoo M., Tahir S.B.U.D., Jalal A., Kim K. (2021). Monitoring real-time personal locomotion behaviors over smart indoor-outdoor environments via body-worn sensors. IEEE Access.

[40] Halim N. (2022). Stochastic recognition of human daily activities via hybrid descriptors and random forest using wearable sensors. Array.

[41] Ghadi Y.Y., Javeed M., Alarfaj M., Shloul T.A., Alsuhibany S.A., Jalal A., Kamal S., Kim D. (2022). MS-DLD: Multi-sensors based daily locomotion detection via kinematic-static energy and body-specific HMMs. IEEE Access.

[42] Koşar E., Barshan B. (2023). A new CNN-LSTM architecture for activity recognition employing wearable motion sensor data: Enabling diverse feature extraction. Eng. Appl. Artif. Intell..

[43] Kobayashi S., Hasegawa T., Miyoshi T., Koshino M. (2023). MarNASNets: Toward CNN Model Architectures Specific to Sensor-Based Human Activity Recognition. IEEE Sens. J..

[44] Wang Y., Xu H., Liu Y., Wang M., Wang Y., Yang Y., Zhou S., Zeng J., Xu J., Li S. (2023). A Novel Deep Multifeature Extraction Framework Based on Attention Mechanism Using Wearable Sensor Data for Human Activity Recognition. IEEE Sens. J..

[45] Dua N., Singh S.N., Semwal V.B. (2021). Multi-input CNN-GRU based human activity recognition using wearable sensors. Computing.

[46] Imran H.A., Ikram A.A., Wazir S., Hamza K. EdgeHARNet: An Edge-Friendly Shallow Convolutional Neural Network for Recognizing Human Activities Using Embedded Inertial Sensors of Smart-Wearables. Proceedings of the 2023 International Conference on Communication, Computing and Digital Systems (C-CODE).

[47] Zhang J., Liu Y., Yuan H. (2023). Attention-Based Residual BiLSTM Networks for Human Activity Recognition. IEEE Access.

[48] Thakur D., Roy S., Biswas S., Ho E.S.L., Chattopadhyay S., Shetty S. A Novel Smartphone-Based Human Activity Recognition Approach using Convolutional Autoencoder Long Short-Term Memory Network. Proceedings of the 2023 IEEE 24th International Conference on Information Reuse and Integration for Data Science (IRI).

[49] Li S., Li Y., Yun F. Multi-view time series classification: A discriminative bilinear projection approach. Proceedings of the 25th ACM International Conference on Information and Knowledge Management ACM.

[50] Fushiki T. (2011). Estimation of prediction error by using K-fold cross-validation. Stat. Comput..

[51] Fawcett T. (2006). An introduction to ROC analysis. Pattern Recognit. Lett..

